# Ionic Liquids as Alternative Curing Agents for Conductive Epoxy/CNT Nanocomposites with Improved Adhesive Properties

**DOI:** 10.3390/nano13040725

**Published:** 2023-02-14

**Authors:** Lidia Orduna, Itziar Otaegi, Nora Aranburu, Gonzalo Guerrica-Echevarría

**Affiliations:** POLYMAT and Department of Advanced Polymers and Materials: Physics, Chemistry and Technology, Faculty of Chemistry, University of the Basque Country (UPV/EHU), Paseo Manuel de Lardizabal 3, 20018 Donostia-San Sebastián, Spain

**Keywords:** epoxy resin, ionic liquid, carbon nanotube, nanocomposite, curing agent, dispersing agent, mechanical properties, lap shear, conductivity

## Abstract

Good dispersion of carbon nanotubes (CNTs) together with effective curing were obtained in epoxy/CNT nanocomposites (NCs) using three different ionic liquids (ILs). Compared to conventional amine-cured epoxy systems, lower electrical percolation thresholds were obtained in some of the IL-based epoxy systems. For example, the percolation threshold of the trihexyltetradecylphosphonium dicyanamide (IL-P-DCA)-based system was 0.001 wt.%. The addition of CNTs was not found to have any significant effect on the thermal or low-strain mechanical properties of the nanocomposites, but it did improve their adhesive properties considerably compared to the unfilled systems. This study demonstrates that ILs can be used to successfully replace traditional amine-based curing agents for the production of electrically conductive epoxy/CNT NCs and adhesives, as a similar or better balance of properties was achieved. This represents a step towards greater sustainability given that the vapor pressure of ILs is low, and the amount needed to effectively cure epoxy resins is significantly lower than any of their counterparts.

## 1. Introduction

Epoxy resins are widely used due to their excellent mechanical and adhesive properties. One way to further improve these properties and acquire others—such as electrical conductivity—is by adding carbon-based nanofillers such as carbon nanotubes (CNTs). CNTs are an allotropic form of carbon made up of one (SWCNT) or multiple (MWCNT) one-atom-thick graphene sheets that are rolled into hollow cylinders with a nanometric diameter. They present outstanding mechanical and electrical properties. However, the nanofiller must be properly dispersed for the nanocomposites (NCs) to obtain a good balance of properties.

Epoxy resin/CNT NCs cured with traditional curing agents, such as amines, anhydrides, polyamides and imidazoles, have been widely studied [1,2,3,4,5]. It is well known that the addition of CNTs improves low-strain mechanical properties [1,2,6,7,8,9,10]. However, high concentrations of CNTs can cause aggregates to form [1,2,3], thus limiting their effectiveness as reinforcing agents. Regarding their adhesive properties, while a small number of CNTs can increase lap shear strength, larger quantities can cause more and larger aggregates to form, thus conferring a negative effect on their adhesive properties [4,5,11].

In fact, the strong propensity of CNTs to form bundles and aggregates (due to π–π stacking and Van der Waals forces of attraction) is one of their main drawbacks, as it limits their potential for improving mechanical and adhesive properties. In recent years, the use of ionic liquids (ILs) has been reported to enhance the dispersion of CNTs, thereby improving the properties of NCs based on different thermoplastic [12,13,14] and thermosetting [15,16,17,18] polymers as a result. This is because ILs interact with CNTs through cation–π interactions [19,20] and interrupt the π–π forces of attraction [21]. Improved dispersion levels [13,22,23,24,25] have led to both increases in electrical conductivity [12,25,26,27] and decreases in the percolation threshold [12,25,27].

ILs are generally defined as salts that melt at temperatures below 100 °C. They have a low vapor pressure, are ionically conductive, and are thermally and chemically resistant. Due to these properties, they are used in a wide variety of applications including as solvents in synthesis [28], electrolytes in batteries [29], and as catalysts [30]. In the field of polymer science and technology, they are also used as compatibilizers for immiscible blends [31,32,33], as plasticizers [34,35], as curing agents for epoxy resins [36,37,38,39,40,41,42,43,44] and, as previously mentioned, as dispersing agents for nanofillers.

Regarding the role of ILs as curing agents in epoxy-based systems (as we reported in previous works [45,46]), imidazolium- [38,39,40,41] and phosphonium- [36,37,42,43,44,45,46] based ILs are effective substitutes for traditional volatile and toxic curing agents. In addition, as they act as initiators rather than as comonomers [42], less IL is required to cure the epoxy resin effectively. Several papers have been published on the effect of ILs (as dispersing and/or curing agents) on the mechanical, electrical, and/or adhesive properties of epoxy/CNT systems [6,7,8,9,15,16,18,36,37,47,48].

Santos et al. [15] investigated the properties of epoxy/MWCNT NCs with the IL tributyl(ethyl)-phosphonium diethylphosphate used as the curing and dispersing agent. They observed that the mixing procedure significantly affected the degree of dispersion of the CNTs. In their study, the percolation threshold (p_c_) was reached at 0.016 vol% when 10 phr of IL was used and at 0.047 vol% when 30 phr of IL was used. Using a different phosphonium-based IL (tri(hexyl)tetradecyl phosphonium bis(2,4,4-trimethylpentyl) phosphinate) as the dispersing and curing agent in epoxy/MWCNT NCs, Soares et al. [36] and Maka et al. [37] achieved p_c_-s of between 0.25 and 0.5 phr MWCNTs [36] and below 0.25 wt% [37], respectively.

Alves et al. [16] and Lopes Pereira et al. [48] studied the role of the IL 1-butyl-3-methyl-imidazolium tetrafluoroborate as a dispersing agent in epoxy NCs containing 1 phr MWCNTs and cured with a commercial hardener. They observed that the electrical conductivity of the NC containing IL was three orders of magnitude greater than the NC without IL [48]. However, the addition of IL effected a decrease in the lap shear strength (from 20.4 MPa (the epoxy/CNT) to 14.5 MPa (the epoxy/CNT/IL)) caused by its lubricating effect [16]. Hameed et al. [9] attained individually dispersed MWCNTs using the same IL (1-butyl-3-methylimidazolium tetrafluoroborate) as the dispersing and co-curing agent (with a commercial amine). The authors reported that the diameter of the CNTs in the NCs containing the IL was larger, suggesting that the CNTs were wrapped in or covered by the IL. The addition of 0.5 wt% CNTs caused a 13% increase in Young’s modulus and a 23% increase in tensile strength.

Waters et al. [8,18] and Throckmorton et al. [47] obtained epoxy/SWCNT composites using an IL (1-ethyl-3-methylimidazolium dicyanamide) as the initiator and dispersing agent. The authors reported a percolation threshold of 0.01 wt% SWCNTs for the system containing the IL, which was significantly lower than that of the corresponding traditional amine-cured system (0.4 wt%) [18,47]. Indeed, when the processing conditions were further optimized, an even lower percolation threshold (0.005 wt%) was achieved [18]. Regarding the system’s mechanical properties, the addition of 0.1 wt% SWCNTs to the epoxy/IL system led to a 9% improvement in Young’s modulus, which the authors attributed to the good dispersion of the nanotubes [8].

Kleinschmidt et al. [6] analyzed the effect of the IL 1-n-butyl-3-methylimidazolium bis(trifluoromethanesulfonyl)imide as the dispersing agent of an epoxy-based NC containing 0.1 wt% MWCNTs cured with two amines. They observed that while the addition of the CNTs led to a decrease in the tensile strength of the neat epoxy resin, the addition of the IL led to an improvement, which the authors attributed to the improved adhesion between the CNTs and the epoxy resin. Finally, the tensile moduli of the epoxy resin and of the epoxy/CNT and epoxy/CNT/IL nanocomposites were all similar.

Gholami et al. [7] also analyzed the dispersion efficiency of different choline chloride-based ILs in epoxy systems containing 0.3 wt% MWCNTs and cured with commercial hardeners. They observed that the addition of the glycerol and choline chloride-based ILs improved the electrical conductivity of the epoxy/CNT system by three orders of magnitude (from 10^−8^ S/cm to 10^−5^ S/cm). They attributed this to the ability of the IL to arrange the electrically conductive CNTs in the matrix and form a more complete conductive network. The enhanced dispersion of the CNTs also led to a 12% improvement in the tensile strength (from 64 MPa to 72 MPa) of the epoxy/CNT NCs.

However, while the dispersive effect of ILs in epoxy/CNT systems has been discussed quite widely in the literature, the role of ILs as effective curing agents has received less attention. Moreover, to the best of our knowledge, no studies have been conducted on the effect of different ILs and CNT concentrations on the final mechanical, electrical, and adhesive properties of epoxy NCs. Therefore, in this study, three different ionic liquids were selected (based on a previous study [46]) and used as curing/dispersing agents for epoxy/CNT NCs, with the objective of optimizing the performance of epoxy NCs without using traditional, volatile curing agents. With this aim in mind, the nanostructure and the thermal, electrical, mechanical, and adhesive properties of the NCs were determined and compared.

## 2. Materials and Methods

### 2.1. Materials

The ionic liquids used in this study were as follows: (a) trihexyltetradecylphosphonium bis(2,4,4-trimethylpentyl)phosphinate (IL-P-TMPP), (b) 1-ethyl-3-methylimidazolium dicyanamide (IL-I-DCA) from Sigma Aldrich, and (c) trihexyltetradecylphosphonium dicyanamide (IL-P-DCA) from IoLiTec-Ionic Liquid Technologies GmbH. Table 1 shows the structures and properties of all three. The epoxy resin employed was a diglycidyl ether of bisphenol A (DGEBA) (Nazza, Eurotex) (epoxy equivalent: 186 g; density (at 20 °C): 1.17 g/cm^3^; viscosity (at 25 °C): 11,500–13,500 mPa·s). A traditional amine-based curing agent, 2,2′-dimethyl-4,4′-methylenebis(cyclohexylamine) (Aradur) (Huntsman), was used as a reference. The carbon nanotubes used in this work were of the Nanocyl NC7000 series (L = 1.5 µm, D = 9.5 nm, 250–300 m^2^/g surface area, 90% purity) supplied by Nanocyl.

### 2.2. Preparation of Samples

The epoxy resin was previously degassed in a vacuum oven at 80 °C for one hour. For the reference unfilled epoxy/IL and epoxy/Aradur systems, 10 phr of IL (selected as the optimum content based on our previous work [46]) and the stoichiometric concentration of the amine, respectively, were added to the epoxy resin, and the resulting mixtures were mechanically mixed at 50 °C using a Heidolph RZR2000 digital rod stirrer until completely homogeneous mixtures were obtained. They were then poured into the corresponding molds or between substrates and were cured according to the curing protocols shown in Table 2.

**Figure 1:**
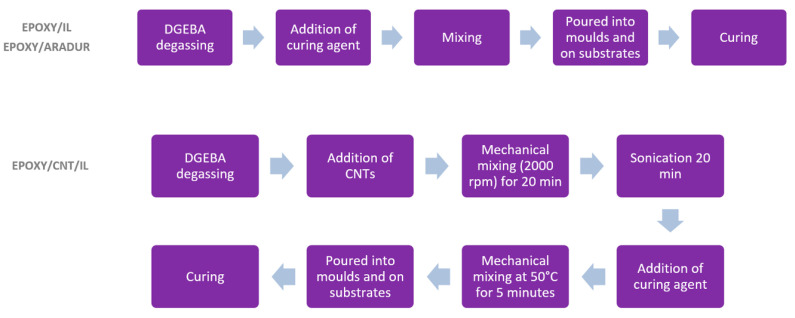
Experimental flowchart illustrating the steps used in the preparation of the samples for the different systems in the study.

For the epoxy/CNT/IL systems, after degassing the DGEBA, the CNTs were added at concentrations ranging from 0 to 0.25% and mechanically mixed at 2000 rpm for 20 min using a EUROSTAR power control-visc digital stirrer. They were then ultrasonicated in a Hielscher UP400s at an amplitude of 100% for 20 min. Next, the IL (10 phr) was added and mechanically mixed at 50 °C for 5 min using a Heidolph RZR2000 digital rod stirrer. Finally, the mixture was poured into molds or placed between lap shear test substrates and the appropriate curing protocol was applied (Table 2). Figure 1 shows the experimental flowchart.

### 2.3. Characterization

#### 2.3.1. Thermal Properties

Dynamic mechanical analysis (DMA) was used to determine the glass transition temperature and to calculate the crosslinking density of the systems. Rectangular, nominally sized specimens measuring 17.5 × 6.0 × 2.0 mm^3^ were tested using a TA Q800 viscoelastometer in single-cantilever bending mode, with a frequency of 1 Hz and an amplitude of 15 μm. The heating rate was set at 4 °C/min and the temperature interval ranged from −100 °C to 250 °C. One specimen was tested per composition. The elasticity theory (Equation (1)) was used to calculate the crosslinking density (*ν_e_*):

(1) $$\nu_{e}=\frac{E_{r}}{3RT_{r}}$$

where *E_r_* is the storage modulus in the rubbery state at a reference temperature (*T_r_* = 245 °C) and *R* is the ideal gas constant (8.314 J/mol·K).

#### 2.3.2. Nanostructure

The nanostructure and the dispersion level of the CNTs were analyzed by transmission electron microscopy (TEM). A Tecnai G2 20 Twin microscope was used at an accelerating voltage of 200 kV. The samples were cut at a 45° angle using a Leica EM UCG ultramicrotome with a diamond blade.

#### 2.3.3. Electrical Properties

The electrical conductivity through the sample was measured using a digital Keithley 6487 picoammeter on circular samples (Ø70 mm × 2 mm thick), to which 1 V was applied for 1 min. The electrical conductivity (*σ*) was calculated using Equation (2):

(2) $$\sigma =\frac{1}{\rho }=\frac{thickness\;\left(\mathrm{cm}\right)\times I}{22.9\times V}\;\;\;\left(S/\mathrm{cm}\right)$$

where *ρ* is the resistivity, *V* is the voltage applied, *I* is the intensity of the current, and 22.9 is the geometrical constant (specific area between electrodes). Three measurements were made for each reported value.

The percolation threshold was calculated using Equation (3):

(3) $$\sigma \left(p\right)=B{\left(p-p_{c}\right)}^{t}$$

where *σ(p)* is the conductivity, *B* is a constant, *t* is the critical exponent, *p* is the nanofiller concentration, and *p_c_* is the percolation concentration. The experimental data were fitted by plotting log(*σ*) vs. log(*p* − *p_c_*) and increasing *p_c_* until the best linear fit was obtained.

#### 2.3.4. Mechanical Properties

Three-point bending tests were carried out in an Instron 5569 universal testing machine. The span was set at 64 mm with a crosshead speed of 2 mm/min. At least 5 specimens were tested for each composition.

The flexural modulus (*E_f_*), the flexural strength (*σ_F_*), and the deformation at break (*ε_F_*) were calculated according to the ISO 178 standard, using Equations (4), (5) and (6), respectively:

(4) $$E_{f}=\frac{FL^{3}}{4sbh^{3}}$$

(5) $$\sigma_{F}=\frac{3F_{max}L}{2bh^{2}}$$

(6) $$\varepsilon_{F}\;\left(\%\right)=\frac{6sh}{L^{2}}\times 100$$

where *F* is the load, *F_max_* is the maximum load, *L* is the span, *s* is the degree of deflection, and *b* and *h* are the width and thickness of the specimen, respectively.

Notched Charpy impact tests were carried out using a Ceast 6548/000 pendulum with an impactor of 2 J. Notched specimens with a radius of 0.25 mm and a depth of 2.54 mm were used. At least 8 specimens were tested for each composition.

#### 2.3.5. Adhesive Properties

The adhesive properties were studied by performing lap shear strength tests according to the ASTM D-1002 standard. The substrates—aluminum 2021-T351 alloy sheets measuring 100 mm × 25 mm × 1.6 mm—were purchased from Rocholl GmbH, and the tested adhesion area measured 12.5 mm × 25 mm. An Instron 5569 universal testing machine in tensile mode was used, employing a constant speed of 1 mm/min. The lap shear strength was calculated by dividing the maximum force by the adhesion area. For each reported value, 10 specimens were tested.

## 3. Results and Discussion

### 3.1. Thermal Properties

**Figure 2:**
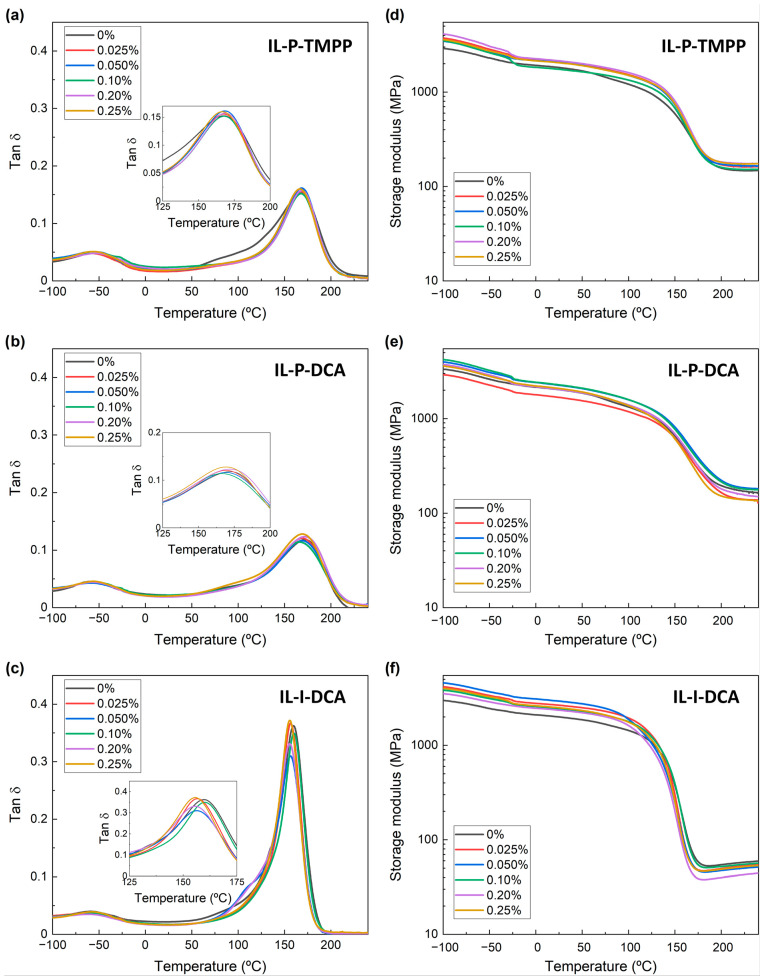
Tan δ (**a**–**c**) and storage modulus (**d**–**f**) obtained by DMTA for IL-P-TMPP (**a**,**d**), IL-P-DCA (**b**,**e**), and IL-I-DCA (**c**,**f**) epoxy/IL NCs at different CNT fractions.

Figure 2 shows the tanδ and the storage modulus vs temperature curves of the samples cured with the different ILs at different CNT concentrations. The main peak of the tanδ curves, which appears at high temperatures (shown in the inset), indicates the glass transition temperature (T_g_). The maximums of the peaks for the IL-based systems, along with the calculated crosslinking density values, are summarized in Table 3.

As Figure 2 and Table 3 show, neither the glass transition temperature nor the crosslinking density changed significantly upon the addition of the CNTs. In fact, both higher [49,50,51] and lower [52,53,54] T_g_ values have been reported when CNTs were added to epoxy resins, so there is no general consensus in the literature regarding the impact of CNTs on the T_g_ of conventionally cured epoxy/CNT systems. Decreases in T_g_ are generally attributed to the steric hindrance of the CNTs towards the curing reaction [54,55], which gives rise to samples with less cross-linkage. By contrast, increases in T_g_ are usually linked to decreases in the mobility of the macromolecular chains caused by the presence of CNTs. This effect is more pronounced the greater the degree of dispersion or adhesion, i.e., the two parameters reported to most heavily impact the T_g_ [51]. Regarding amine-cured epoxy systems with ILs as dispersing agents, both increases [9] and decreases [17,48,56] in the T_g_ have been reported. The effect of the presence of ILs is not completely clear in these cases because they are also known to act as effective plasticizers. Both decreases [36] and increases [15,37] in T_g_ have also been reported for epoxy/CNT NCs where the ILs acted as both curing and dispersing agents. The behavior of the epoxy/Aradur system in this study was similar to that of the epoxy/IL systems; the addition of CNTs did not significantly affect the T_g_ or the crosslinking density of the unfilled system (the T_g_ and crosslinking density of the 0.2 wt.% CNT composition were 191 °C and 3090 mol/m^3^, respectively).

### 3.2. Nanostructure

**Figure 3:**
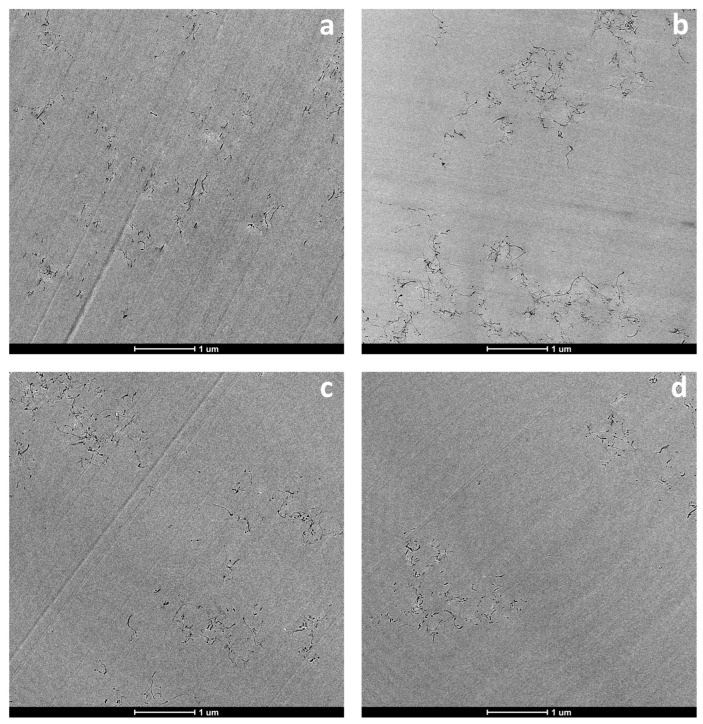
TEM micrographs of the epoxy/IL-P-DCA (**a**), epoxy/IL-P-TMPP (**b**), epoxy/IL-I-DCA (**c**), and the reference epoxy/Aradur systems containing 0.2 wt.% CNT (**d**).

The nanostructures of the epoxy/IL systems were analyzed by transmission electron microscopy (TEM). Figure 3a–c show representative micrographs of the IL-P-DCA, IL-P-TMPP, and IL-I-DCA epoxy/IL systems, respectively, each containing 0.2 wt.% of CNTs. As a reference, Figure 3d shows a representative TEM micrograph of the epoxy/Aradur NC with the same concentration of CNTs. As can be seen in Figure 3a–c, good dispersion of the nanofiller was achieved in the case of all three ILs, with mostly individually dispersed CNTs and far fewer small aggregates. When the different ILs are compared, it is evident that, in Figure 3a, there are fewer and smaller CNT aggregates than those appearing in Figure 3b,c, indicating that the CNTs were best dispersed in the epoxy/CNT/IL-P-DCA system, and more poorly dispersed in the epoxy/CNT/IL-P-TMPP and epoxy/CNT/IL-I-DCA systems. A similar nanostructure was observed in the reference epoxy/CNT/Aradur system (shown here in Figure 3d). As expected, these differences significantly affected the electrical properties of the NCs and are discussed below.

The achievement of good dispersion of CNTs is known to be complicated due to interactions between the sp^2^ orbitals perpendicular to the layers, which lead to the reaggregation of the CNTs. ILs have been reported to be effective in terms of dispersing CNTs, although how they actually execute this is a controversial topic judging from the bibliography contained herein. In the case of imidazolium-based ILs, some authors indicate that the π–cation interaction [19,20] is likely responsible, while others believe the Van der Waals forces-based interactions [21] cause the rupture of the π–π attractive forces between the CNTs. With respect to the phosphonium-based ILs, in the absence of aromatic groups in their structures, π–cation interactions are preferred [57]. Moreover, as these ILs contain cations with long aliphatic chains, a surfactant effect has also been suggested as being responsible for the dispersive effect [15,36,37].

### 3.3. Electrical Properties

**Figure 4:**
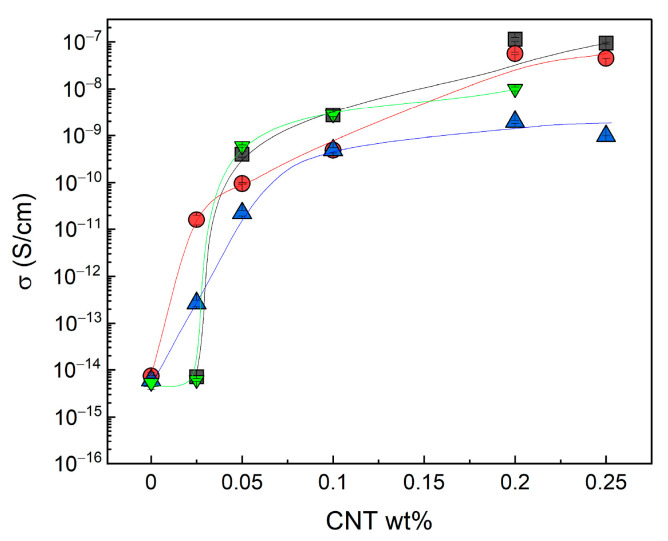
Electrical conductivity of IL-P-TMPP (■), IL-P-DCA (●), IL-I-DCA (▲), and the reference Aradur (▼) epoxy/IL NCs as a function of the CNT concentration.

Figure 4 shows the electrical conductivity vs the CNT content of the different epoxy/IL systems. It can be clearly seen that the addition of CNTs led to a dramatic increase in conductivity in all the epoxy/IL systems. For example, the conductivity values of the 0.2 wt.% CNT composition were 1.13 × 10^−7^, 5.67 × 10^−8^, 1.96 × 10^−9^, and 9.99 × 10^−9^ S/cm for the IL-P-TMPP, IL-P-DCA, IL-I-DCA, and Aradur-based systems, respectively. The percolation thresholds are shown in Table 4, and the fitting parameters *B* and *t* (Equation (3)) along with the conductivity values corresponding to the p_c_ are presented in Table S1. As can be seen, the percolation threshold was reached at very low CNT fractions—0.025 wt.% or lower—in all cases. The IL-P-DCA-cured epoxy NC had the lowest *p_c_* at 0.001 wt.%. This is consistent with the best dispersion levels on the TEM micrographs. Table 4 also shows how the similar CNT dispersion levels of the other two IL-based systems (and for the reference amine-based one) also resulted in similar percolation threshold values. Thus, at low CNT shares, tiny changes in the degree of dispersion can affect the percolation threshold, i.e., the minimum concentration required to create the conductive path necessary for the transition from an insulator to semiconductor to take place.

At high CNT shares, the conductivity values of the systems cured with the phosphonium-based ILs (i.e., IL-P-TMPP and IL-P-DCA) were two orders of magnitude higher than those cured with the imidazolium-based IL (i.e., IL-I-DCA), suggesting that the long chains in the structures of the former two are more effective at preventing the formation of CNT aggregates.

In the literature, a wide range of percolation threshold concentrations have been reported for epoxy/CNT NCs. This variation is probably due to the myriad factors that affect the formation of percolated networks, namely, the aspect ratio of the CNTs. Usually, the reported *p_c_* values do not exceed 1–2 wt.% [58], with most ranging from 0.1 to 1 wt.% [59,60,61]. However, figures as low as 0.04 wt.% [62,63] or 0.001 wt.% [64] have also been reported. Lopes Pereira et al. [48] studied the effect of adding ILs as nanofiller-dispersing agents and reported an increase of three orders of magnitude in electrical conductivity compared to the non-IL system. Regarding the conductivity of the Nanocyl NC7000 CNT-based percolated systems, a wide range of values (from 10^−7^ to 10^−2^ S/cm) can be found in our bibliography depending on the experimental technique or conditions used [15,36,37].

### 3.4. Mechanical Properties

**Figure 5:**
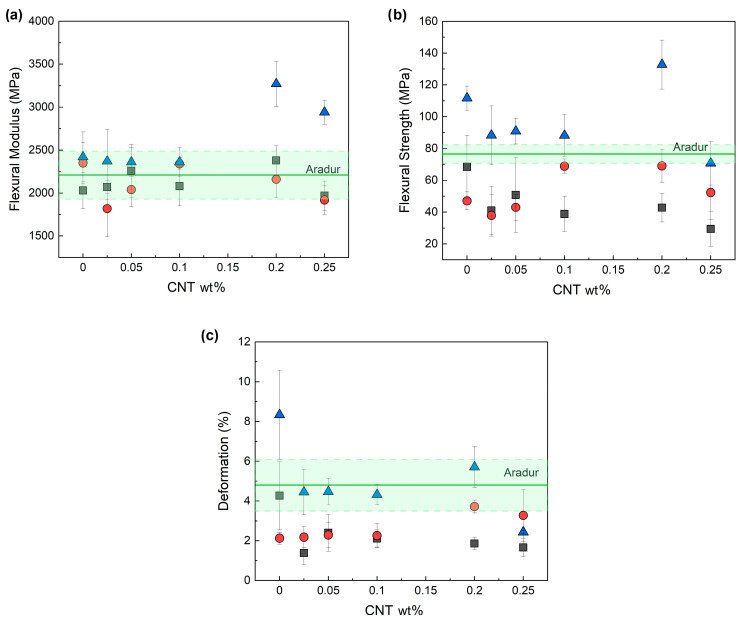
Flexural modulus (**a**), flexural strength (**b**), and deformation at break (**c**) of the epoxy/CNT systems cured with ILs (IL-P-TMPP (■), IL-P-DCA (●), and IL-I-DCA (▲)). The values of the unfilled epoxy resin cured with an amine-based curing agent (Aradur) are also shown as a reference (the solid green line marks the average value, and the shaded area shows the standard deviation).

Figure 5 shows the flexural modulus, flexural strength, and deformation at break of the epoxy/IL NCs as a function of the CNT content. The data for the reference unfilled epoxy/Aradur system have also been included (the green, solid line shows the average value, while the shaded area shows the standard deviation).

As Figure 5a shows, overall, the flexural modulus of the epoxy resin was barely affected by the addition of the CNTs. Slight increases were only observed for the IL-I-DCA-based system at high CNT shares. These results suggest that neither the crosslinking density (Table 3) nor the degree of dispersion of the CNTs (Figure 3a–c) in these systems are directly related to their mechanical properties. Similar results were obtained in the reference epoxy/Aradur system, given that the addition of 0.2 wt.% CNTs did not lead to significant changes in the flexural modulus (2270 MPa) or in flexural strength (56.0 MPa). This is further discussed below.

No significant trends in flexural strength were observed in any of the systems (Figure 5b). As with the flexural modulus, the IL-I-DCA-cured samples with a high concentration of CNTs scored highest. It is worth noting that the flexural modulus and strength data of some of the compositions are similar or better than those of the amine-cured reference system.

**Figure 6:**
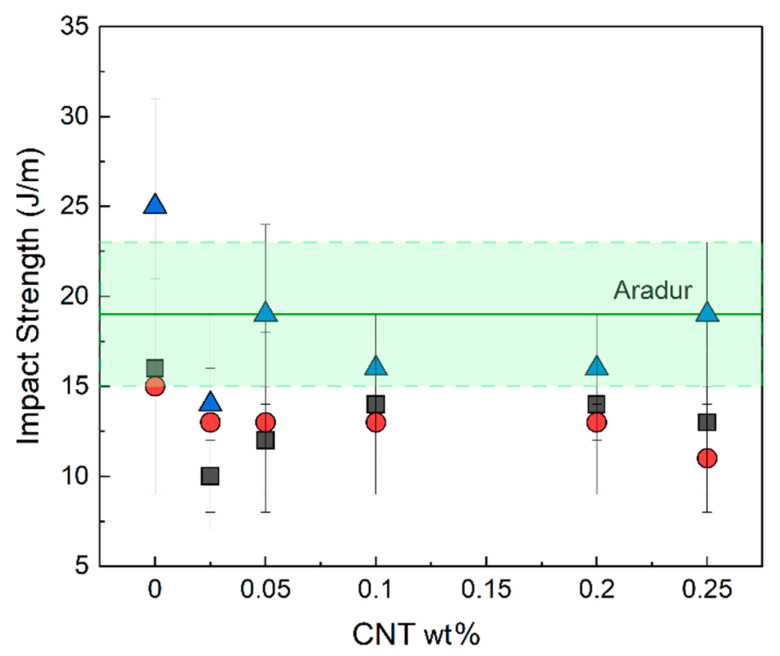
Impact strength of the epoxy/CNT systems cured with ILs (IL-P-TMPP (■), IL-P-DCA (●), and IL-I-DCA (▲)). The value of the unfilled epoxy resin cured with an amine-based curing agent (Aradur) is also shown as a reference (green solid line for the average value and shaded area for the standard deviation).

With respect to high-strain mechanical properties, the deformation at break (Figure 5c) and the impact strength (Figure 6) both decreased when CNTs were added. This was to be expected because nanofillers in general—and CNTs in particular—are known to act as crack initiators and/or stress-concentration points, thus leading to decreases in ductility and toughness [1].

Epoxy resins that have been mechanically improved by adding CNTs have been extensively documented in the literature [1,2]. In amine-cured epoxy/CNT NCs with ILs as dispersing agents, the mechanical properties were further enhanced due to the enhanced levels of dispersion [6,7,9,10]. Nevertheless, the fact that ILs can also act as plasticizers also needs to be taken into account. Accordingly, depending on the amount of IL used, the plasticizing effect may be predominant, causing decreases in low-strain mechanical properties [7].

Few papers on the roles of ILs as both curing and dispersing agents in epoxy systems have examined their mechanical behavior. For those that have, organoclays [65], silica [47], core–shell particles [43], and CNTs [8] were used as nanofillers. In the epoxy/IL/CNT system [8], for example, the authors reported that Young’s modulus increased with the CNT content with respect to the unfilled epoxy/IL-I-DCA system. However, this increase was not significant above 1 wt.% CNTs, which is probably due to the deficient dispersion associated with an excessive number of CNTs.

### 3.5. Adhesive Properties

**Figure 7:**
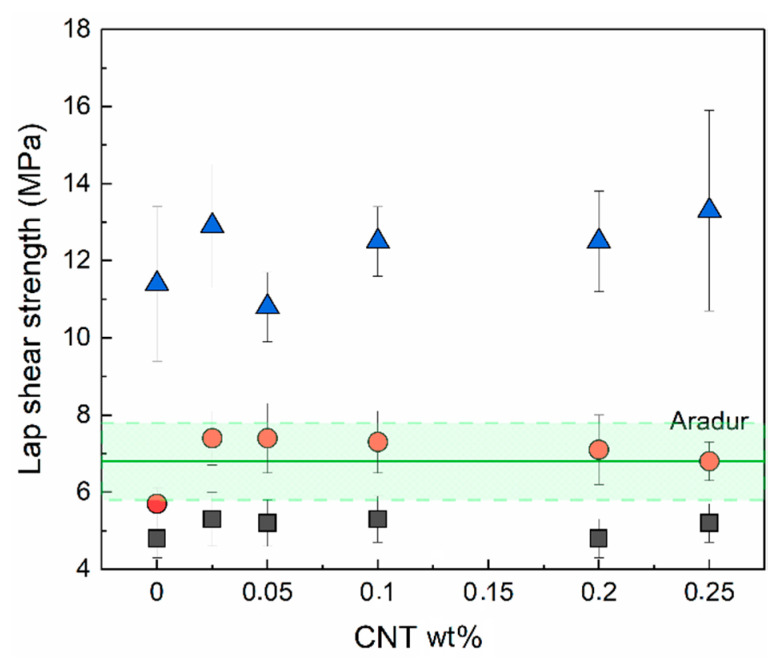
Lap shear strength of the epoxy/CNT systems cured with ILs (IL-P-TMPP (■), IL-P-DCA (●), and IL-I-DCA (▲)). The lap shear strength of the unfilled epoxy resin cured with an amine-based curing agent (Aradur) is also shown as a reference (the green, solid line marks the average value, and shaded area shows the standard deviation).

Figure 7 shows the lap shear strength of the epoxy/IL NCs as a function of their CNT content. The corresponding result for the unfilled epoxy/Aradur system is also shown as a reference. As can be seen, regardless of the IL used, the addition of low concentrations of CNTs led to increases in the lap shear strength compared to the unfilled compositions. However, higher concentrations of CNTs did not lead to higher lap shear strength values. A maximum improvement in adhesive properties (30%) was attained with the IL-P-DCA-cured epoxy system containing 0.025 wt.% CNTs. Similar increases—close to 30% (from 6.8 to 8.8 MPa)—were also observed when 0.2% wt.% CNTs were added to the reference epoxy/Aradur system.

Our findings are consistent with the results reported in the literature for epoxy/CNT systems [4,5,11]. In an epoxy/CNT/amine system, Alves et al. [16] reported that the improvement in the dispersion of CNTs effected by a masterbatch led to enhanced adhesive properties. However, they also observed—in the same study—that even though the dispersion of the CNTs was enhanced when an IL was added, the lap shear strength decreased by over 50% due to the lubricating effect of the IL [13,66].

Considering the results in Figure 7, it is noteworthy that, regardless of the CNT content, all the epoxy/IL-I-DCA NCs showed significantly enhanced adhesive properties compared to both the reference unfilled epoxy/Aradur system and the CNT-filled epoxy/Aradur composition. So, in summary, electrically conductive epoxy adhesives with outstanding adhesive properties were obtained when ILs were used as both the curing and dispersing agents.

## 4. Conclusions

The dual role of ionic liquids (ILs) as effective curing and dispersing agents produced volatile-amine-free epoxy/CNT nanocomposites with a better balance of mechanical, electrical, and adhesive properties. Three different ILs were tested, and all three led to good dispersion of the nanofiller, featuring individually dispersed CNTs as well as some small aggregates. Overall, with a percolation threshold of 0.001 wt.%, the IL-P-DCA system was the most effective.

The addition of CNTs had no effect on the thermal or low-strain mechanical properties of the epoxy/IL systems. However, it did improve the systems’ adhesive properties. The epoxy/IL-P-DCA system containing 0.025 wt.% CNTs improved by 30% and was the best of the three. This work proves that, using very small amounts of CNTs, it is possible to obtain electrically conductive, amine-free epoxy adhesives with similar mechanical properties but greater lap shear strength than the reference amine-cured epoxy system. ILs have a lower vapor pressure, and a significantly lower amount is needed to effectively cure epoxy resins. Therefore, replacing conventional epoxy resin curing agents (amines, anhydrides, etc.) with ILs is a major step forward in the development of more sustainable materials.

## Figures and Tables

**Table 1:** Structures, properties, and abbreviations of the three ILs used in this study.

Abbreviation	Structure	Properties
IL-P-TMPP	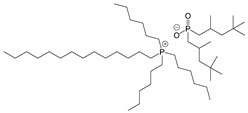	Molecular weight (g/mol): 773.27 Density (20 °C) (g/cm^3^): 0.895
IL-P-DCA	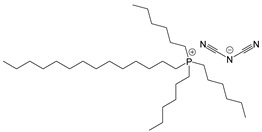	Molecular weight (g/mol): 549.90 Density (20 °C) (g/cm^3^): 0.9
IL-I-DCA	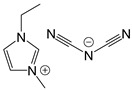	Molecular weight (g/mol): 177.21 Density (20 °C) (g/cm^3^): 1.060

**Table 2:** Curing protocol used for the epoxy/IL and the epoxy/Aradur systems.

Curing Agent	Curing Protocol
IL-P-TMPP	2 h 80 °C/2 h 120 °C/1 h 150 °C/1 h 170 °C
IL-P-DCA	2 h 120 °C/2 h 140 °C/1 h 170 °C
IL-I-DCA	2 h 110 °C/1 h 140 °C/1 h 170 °C
Aradur	2 h 80 °C/2 h 120 °C/1 h 170 °C/1 h 200 °C

**Table 3:** T_g_ and crosslinking density values for the epoxy/IL NCs in the study at different CNT fractions. The T_g_ of the reference epoxy/Aradur system was 189 °C and the crosslinking density was 2790 mol/m^3^.

CNT wt.%	T_g_ (°C)	ν_e_ (mol/m^3^)	CNT wt.%	T_g_ (°C)	ν_e_ (mol/m^3^)	CNT wt.%	T_g_ (°C)	ν_e_ (mol/m^3^)
IL-P-TMPP			IL-P-DCA			IL-I-DCA		
0	168	11509	0	172	12616	0	160	4625
0.025	168	12610	0.025	169	10,828 *	0.025	157	4310
0.05	169	12660	0.05	169	14,146 *	0.05	156	4015
0.1	168	11847	0.1	166	13,669	0.1	160	4398
0.2	169	13594	0.2	173	11,437	0.2	155	3599
0.25	167	13590	0.25	170	10,448	0.25	155	4158

* Calculated at lower T_r_ due to the instability of the curve at 245 °C.

**Table 4:** Percolation thresholds of the epoxy/IL and epoxy/Aradur NCs.

Curing Agent	*p_c_* (CNT wt.%)
IL-P-TMPP	0.025
IL-P-DCA	0.001
IL-I-DCA	0.02
Aradur	0.025

## Supplementary Materials

The following are available online at https://www.mdpi.com/article/10.3390/nano13040725/s1, Table S1: Fitting parameters (*B* and *t*) and conductivity values corresponding to the p_c_ of the epoxy/CNT/IL NCs cured with IL-P-TMPP, IL-P-DCA and IL-I-DCA and of the reference epoxy/CNT/Aradur systems.
