# Supplementary material for: Ionic Liquids as Alternative Curing Agents for Conductive Epoxy/CNT Nanocomposites with Improved Adhesive Properties
Source: Nanomaterials (Basel). 2023 Feb 14;13(4):725. doi: 10.3390/nano13040725 (PMC9966306; doi:10.3390/nano13040725)
Supplement: Supplementary file 1 [file nanomaterials-13-00725-s001.zip › nanomaterials-2198966-supplementary.pdf]

Table S1. Fitting parameters (*B* and *t*) and conductivity values corresponding to the p_c of the epoxy/CNT/IL NCs cured with IL-P-TMPP, IL-P-DCA and IL-I-DCA and of the reference epoxy/CNT/Aradur systems.

System	*B (S/cm)*	*t*	*σ(p_c) (S/cm)*
IL-P-TMPP	$6.66 \cdot 10^{-6}$	2.72	$7.16 \cdot 10^{-15}$
IL-P-DCA	$7.91 \cdot 10^{-6}$	3.67	$6.52 \cdot 10^{-13}$
IL-I-DCA	$7.44 \cdot 10^{-8}$	2.32	$2.15 \cdot 10^{-13}$
Aradur	$1.22 \cdot 10^{-7}$	1.45	$6.09 \cdot 10^{-15}$
